# Ablation of Keratan Sulfate Accelerates Early Phase Pathogenesis of ALS

**DOI:** 10.1371/journal.pone.0066969

**Published:** 2013-06-25

**Authors:** Kenichi Hirano, Tomohiro Ohgomori, Kazuyoshi Kobayashi, Fumiaki Tanaka, Tomohiro Matsumoto, Takamitsu Natori, Yukihiro Matsuyama, Kenji Uchimura, Kazuma Sakamoto, Hideyuki Takeuchi, Akihiro Hirakawa, Akio Suzumura, Gen Sobue, Naoki Ishiguro, Shiro Imagama, Kenji Kadomatsu

**Affiliations:** 1 Department of Biochemistry, Nagoya University Graduate School of Medicine, Nagoya, Japan; 2 Department of Orthopedics, Nagoya University Graduate School of Medicine, Nagoya, Japan; 3 Department of Neurology, Nagoya University Graduate School of Medicine, Nagoya, Japan; 4 Department of Health and Nutrition, Yamanashi Gakuin University, Kofu, Japan; 5 Department of Orthopedics, Hamamatsu University School of Medicine, Hamamatsu, Japan; 6 Department of Neuroimmunology, Research Institute of Environmental Medicine, Nagoya University, Nagoya, Japan; 7 Center for Advanced Medicine and Clinical Research, Nagoya University Graduate School of Medicine, Nagoya, Japan; Graduate School of Pharmaceutical Sciences, The University of Tokyo, Japan

## Abstract

Biopolymers consist of three major classes, i.e., polynucleotides (DNA, RNA), polypeptides (proteins) and polysaccharides (sugar chains). It is widely accepted that polynucleotides and polypeptides play fundamental roles in the pathogenesis of neurodegenerative diseases. But, sugar chains have been poorly studied in this process, and their biological/clinical significance remains largely unexplored. Amyotrophic lateral sclerosis (ALS) is a motoneuron-degenerative disease, the pathogenesis of which requires both cell autonomous and non-cell autonomous processes. Here, we investigated the role of keratan sulfate (KS), a sulfated long sugar chain of proteoglycan, in ALS pathogenesis. We employed ALS model SOD1^G93A^ mice and GlcNAc6ST-1^−/−^ mice, which are KS-deficient in the central nervous system. Unexpectedly, SOD1^G93A^GlcNAc6ST-1^−/−^ mice exhibited a significantly shorter lifespan than SOD1^G93A^ mice and an accelerated appearance of clinical symptoms (body weight loss and decreased rotarod performance). KS expression was induced exclusively in a subpopulation of microglia in SOD1^G93A^ mice, and became detectable around motoneurons in the ventral horn during the early disease phase before body weight loss. During this phase, the expression of M2 microglia markers was transiently enhanced in SOD1^G93A^ mice, while this enhancement was attenuated in SOD1^G93A^GlcNAc6ST-1^−/−^ mice. Consistent with this, M2 microglia were markedly less during the early disease phase in SOD1^G93A^GlcNAc6ST-1^−/−^ mice. Moreover, KS expression in microglia was also detected in some human ALS cases. This study suggests that KS plays an indispensable, suppressive role in the early phase pathogenesis of ALS and may represent a new target for therapeutic intervention.

## Introduction

Amyotrophic lateral sclerosis (ALS) is a life-threatening neurodegenerative disease specific to upper and lower motoneurons. Extensive studies on ALS models have revealed that both cell-autonomous and non-cell-autonomous mechanisms are required for the onset and progression of this disease [1], [2], [3], [4]. Among non-cell-autonomous mechanisms, microglia are thought to be important for the progression [2]. Although many important proteins, such as superoxide dismutase 1 (SOD1), TDP43, FUS/TLS, C9ORF72, profilin 1 and optineurin, have been identified [5], the pathogenesis of ALS is not fully understood, and the development of preventive and/or therapeutic measures for this disease is still an unmet goal. In particular, the significance of sugar chains has been little studied in this disease.

Although there is still debate in regard to the proper categorization of microglia, it is widely accepted that activated microglia exert dual functions, i.e., pro-inflammatory and anti-inflammatory functions [6]. Accordingly, activated microglia are sometimes categorized as M1 or M2 microglia. Thus, upon activation, M1 microglia express pro-inflammatory molecules, such as NOX2, IL-1beta and TNF-alpha, as well as cell surface markers, including CD86 [7], [8], [9]. On the other hand, M2 microglia express a different set of cytokines and molecular markers that are associated with anti-inflammatory functions, and which include IL-4, CD206, Ym1 and arginase1 [7], [8], [10], [11]. Although this categorization might be an oversimplification, the *in vivo* status of activated microglia is probably on a continuum between these two extreme states. Thus, microglia can be polarized into an activation state that is intermediate between a pro-inflammatory and an anti-inflammatory state.

Chondroitin sulfate (CS), a long sugar chain (named glycosaminoglycan) of proteoglycans, functions as a potent inhibitor for axonal regeneration/sprouting and synaptic plasticity [12], [13]. Keratan sulfate (KS) is another glycosaminoglycan, and is recognized by the KS-specific antibody 5D4. KS is composed of repeating disaccharide units of 3Galβ1–4GlcNAcβ1, where the C6 position of the GlcNAc residue is always sulfated, while that of Gal is sometimes sulfated. GlcNAc6ST-1 mediates sulfation of GlcNAc, which is an essential step for KS biosynthesis [14]. Therefore, GlcNAc6ST-1-deficient (GlcNAc6ST-1^−/−^) mice show loss of KS in the central nervous system (CNS) [15]. Interestingly, this loss seems to be specific to the CNS, since other organs such as cornea and cartilage express KS in GlcNAc6ST-1^−/−^ mice (Uchimura et al., unpublished data) [15]. We recently found that 5D4-reactive KS inhibits axonal regeneration/sprouting, and its inhibitory activity is as strong as that of CS [16], [17]. On the other hand, the roles of glycosaminoglycans in neurodegenerative diseases have been little studied. Here, we demonstrate an unexpected role of KS in the early phase pathogenesis of ALS.

## Results

### KS was Induced in the Spinal Cord of SOD1^G93A^ Mice

**Figure 1:**
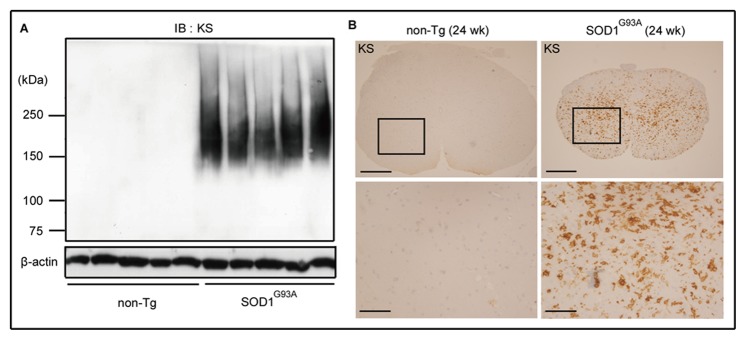
KS expression was induced in the spinal cords of SOD1^G93A^ mice at end stage. (A) Lumbar spinal cord lysates (24 weeks of age) were subjected to immunoblotting using anti-KS antibody (5D4) (n = 5). β-actin was used as the internal loading control. (B) Lumber spinal cord sections obtained from SOD1^G93A^ mice and their age-matched non-Tg littermates were stained with anti-KS antibody. The lower panels are the highly magnified images of the regions marked with squares. Bars, 200 µm in upper panels; 50 µm in lower panels.

To investigate the biological significance of KS in ALS, we employed ALS model SOD1^G93A^ mice. SOD1^G93A^ mice in the end stage (24 weeks after birth) and their age-matched non-Tg littermates were compared for KS expression by immunoblot analysis using the KS-specific antibody 5D4. KS was not expressed in the lumbar spinal cords of non-Tg mice, but was remarkably induced in SOD1^G93A^ mice (Figure 1A). As KS is a long sugar chain, KS-bearing proteoglycans appear as smear bands in immunoblots, as shown in Figure 1A. Immunohistochemical analysis confirmed that KS expression was strikingly induced and distributed throughout the whole area, particularly in the gray matter, in SOD1^G93A^ mice (Figure 1B).

### KS Expression Began in the Early Phase of Disease Before Body Weight Loss

SOD1^G93A^ mice began to lose body weight around 120 days (17–18 weeks) after birth (data not shown). Diseased mice progressively manifested motor paralysis, and reached the end stage at around 24 weeks. It is important to determine when KS starts to emerge in relation to the progression of disease. To this end, we examined the time course of KS expression.

**Figure 2:**
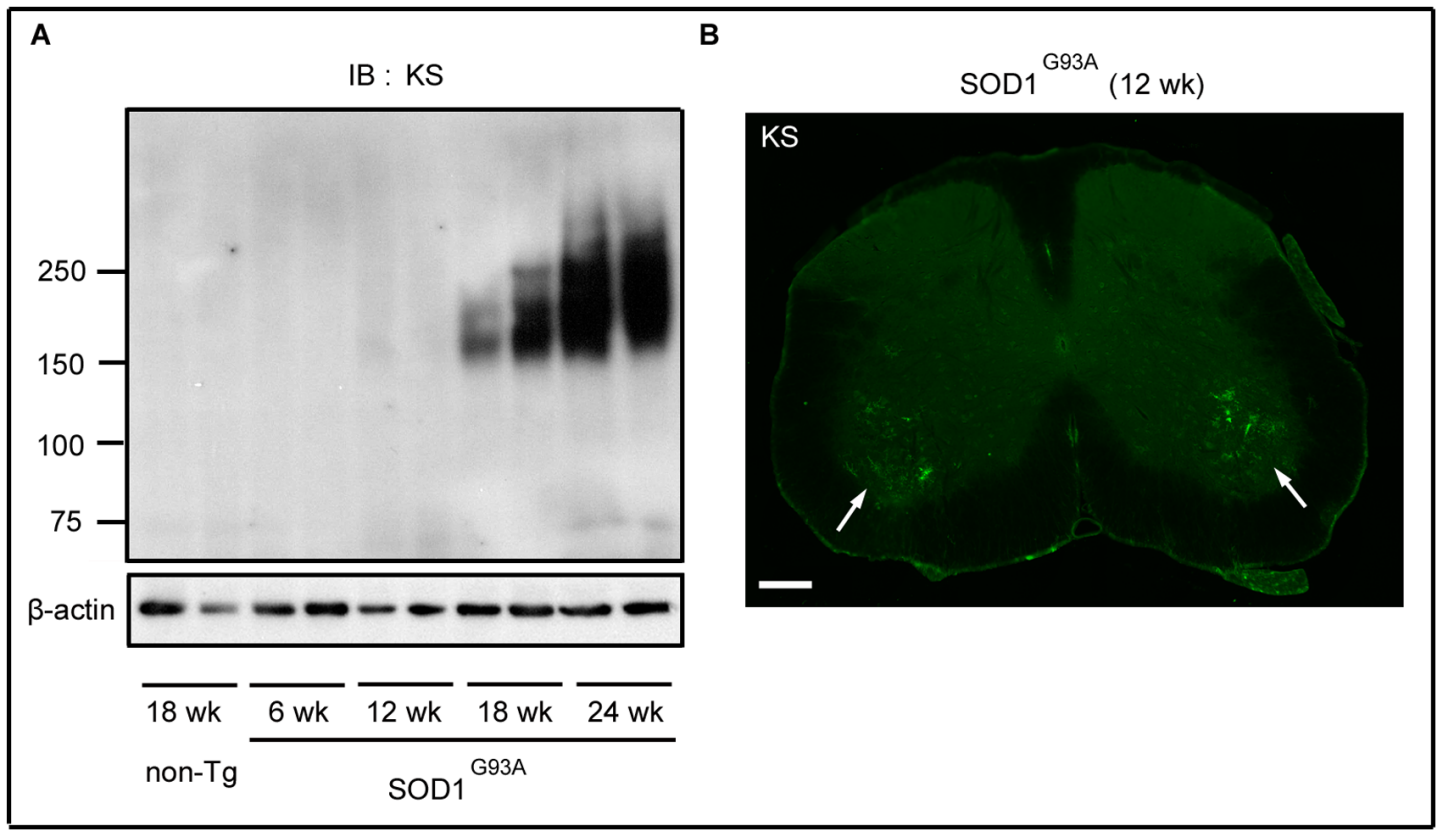
KS expression began before body weight loss. (A) The lysates of the SOD1^G93A^ lumbar spinal cords at 6, 12, 18, and 24 weeks of age were subjected to immunoblotting using the anti-KS antibody 5D4. β-actin was used as the internal loading control. A faint expression of KS was detected at 12 weeks. (B) The SOD1^G93A^ lumber spinal cord section at 12 weeks was stained by anti-KS antibody. KS immunoreactivity was observed in the ventral horn. Bar, 200 µm.

An apparent expression was detected at 18 weeks in the lumbar spinal cord of SOD1^G93A^ mice in immunoblot analysis, although a faint expression of KS was detected at 12 weeks (Figure 2A). The expression increased along with the disease progression (Figure 2A). Notably, immunohistochemical analysis revealed that KS was expressed at 12 weeks in the ventral horn of the spinal cord (Figure 2B, arrows), where motoneurons exist. Thereafter, the distribution of expression expanded throughout the whole area of the spinal cord (Figure 1B). Therefore, the fact that only a faint band was detected at 12 weeks in the immunoblotting analysis was probably due to the restricted localization of KS expression at this stage.

### A Subpopulation of Microglia Expressed KS

**Figure 3:**
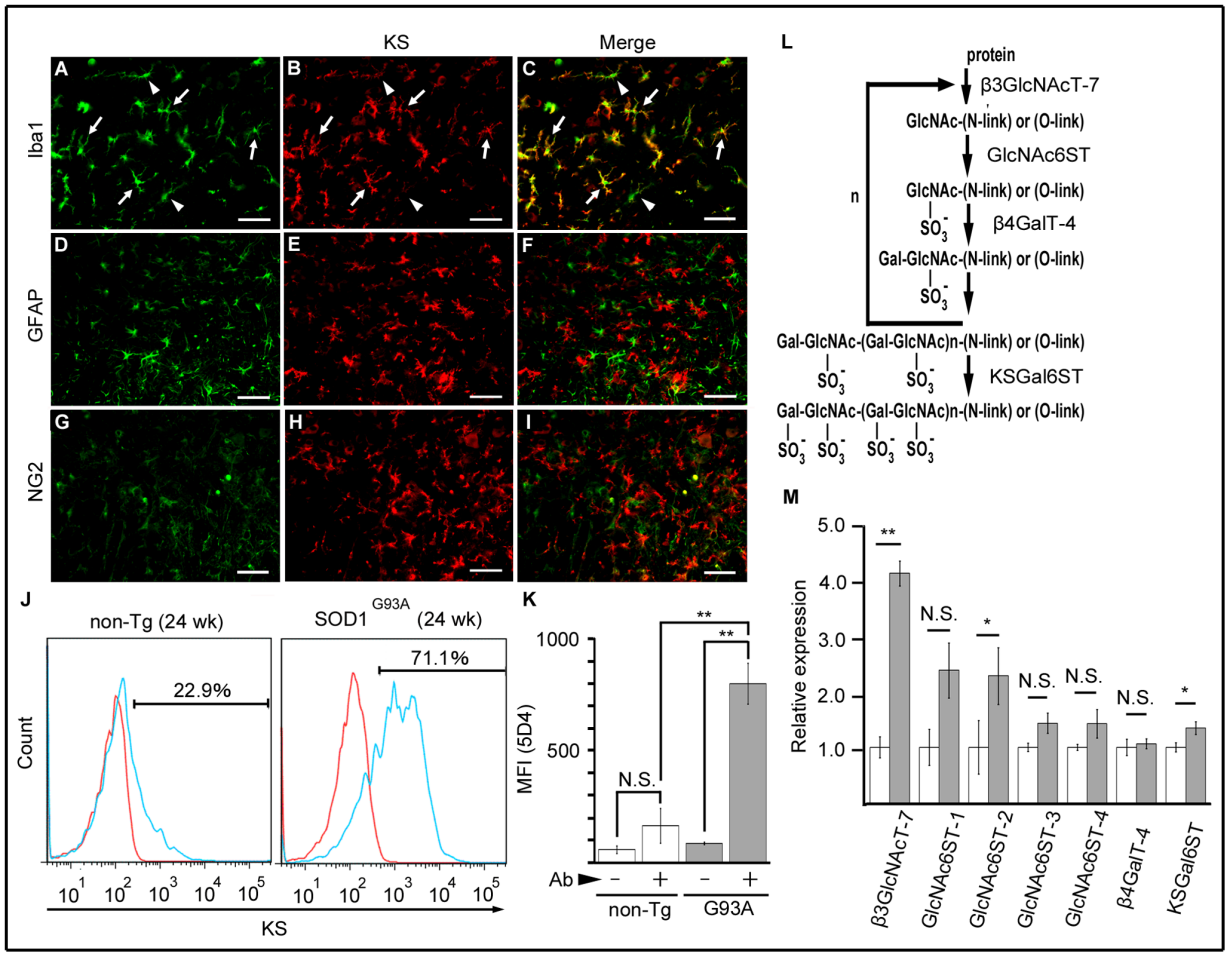
KS was expressed in a subpopulation of microglia. (A-I) The SOD1^G93A^ mice lumbar spinal cords at 18 weeks were stained by anti-KS (B, E, H), anti-Iba1 (microglia marker; A–C), anti-GFAP (astrocyte marker; D–F), and anti-NG2 (oligodendrocyte precursor marker; G–I) antibodies. Merged images are shown in C, F and I. Bars, 50 µm. Arrows, KS^+^Iba1^+^ cells; arrowheads, KS^-^Iba1^+^ cells. (J) A representative profile of KS expression in CD11b^+^ cells. The red line indicates a negative control that does not contain the primary antibody, and the blue line indicates a stained sample. (K) The quantitative data of mean fluorescence intensity (MFI) of KS (n = 3). Error bars, SE. **p<0.01. N.S., not significant. (L) The biosynthesis of KS. (M) mRNA expression of the enzymes involved in KS biosynthesis was examined by quantitative RT-PCR using SOD1^G93A^ (gray columns) and their age-matched non-Tg (white columns) mice at 24 weeks. GAPDH was used as the internal control. Error bars, SE. ** p<0.01, * p<0.05 (n = 4). N.S., not significant.

To determine which cells expressed KS, double immunofluorescence was performed for KS and cell-specific markers on the lumbar spinal cord of SOD1^G93A^ mice at 18 weeks. Colocalization was observed in a subpopulation of Iba1-positive cells (microglia), i.e., both KS-positive and KS–negative cells were found among the Iba1-positive cells (Figure 3A–C). In contrast, no colocalization was detected with glial fibrillary acidic protein (GFAP) (astrocyte; Figure 3D–F), NG2 (oligodendrocyte precursor cell; Figure 3G–I), or MAP2 (neuron) (data not shown). These data suggested that a subpopulation of microglia expressed KS.

We also enriched microglia from the spinal cords of mice at 24 weeks with CD11b affinity beads, and examined their KS expression. The intensity of KS was significantly increased in microglia from SOD1^G93A^ mice as compared with those from non-Tg mice (Figure 3J, K). This enhanced expression of KS in microglia might have been associated with an increased expression of enzymes for KS biosynthesis. To address this question, we next examined the expression levels of these enzymes. The biosynthesis of KS is accomplished through sequential reactions mediated by the enzymes shown in Figure 3L. mRNA expression of these enzymes in the lumbar spinal cord was examined by quantitative RT-PCR for SOD1^G93A^ and age-matched non-Tg mice at 24 weeks. Indeed, a significant increase in mRNA expression was detected for beta3GlcNAcT-7, GlcNAc6ST-2, and KSGal6ST in SOD1^G93A^ mice (Figure 3M). Together, these results support the idea that individual microglial cells increase KS production in SOD1^G93A^ mice.

### KS Expression was Detected in Human ALS

**Figure 4:**
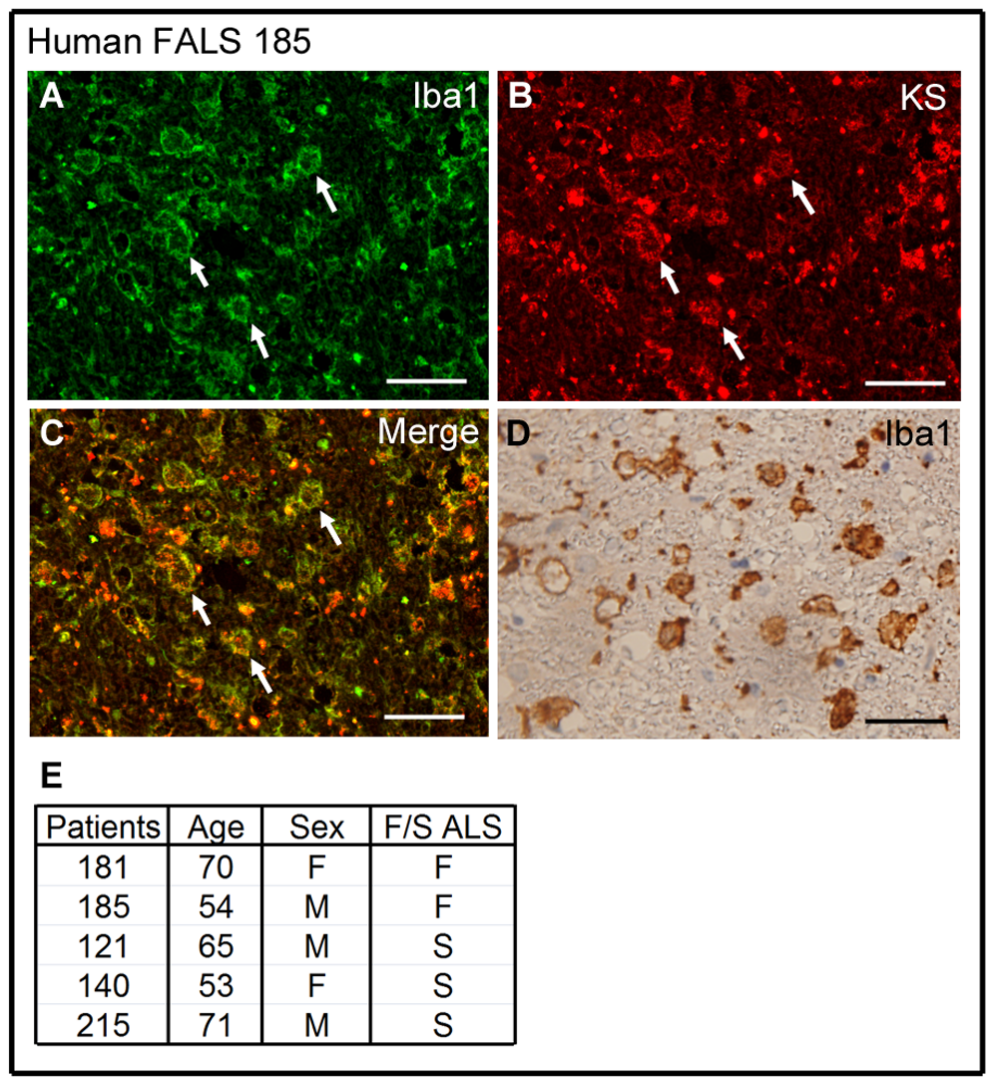
KS expression was detected in human ALS. Human ALS spinal cord sections were stained by anti-Iba1 (A) and anti-KS antibodies (B). Arrows, Iba1^+^KS^+^ cells. Bars, 50 µm. The morphology of microglia was confirmed as anti-Iba1 antibody and DAB staining (D). The information of human ALS samples is shown in Figure 4E.

We next asked whether KS might also be expressed in human ALS. To answer this question, we selected human ALS frozen specimens which showed appropriate Iba1 staining. Notably, we found KS expression in microglia in 5 cases (2 cases of familial ALS and 3 cases of sporadic ALS). This may partly support the idea that our findings in mouse models could be relevant to human ALS, although further large scale clinical studies are needed to evaluate the significance of KS expression in human ALS. A representative case of familial ALS is shown in Figure 4A–D, and the information of human ALS samples is shown in Figure 4E.

### Ablation of KS Expression in Microglia Accelerated the Pathogenesis of ALS in a Mouse Model

**Figure 5:**
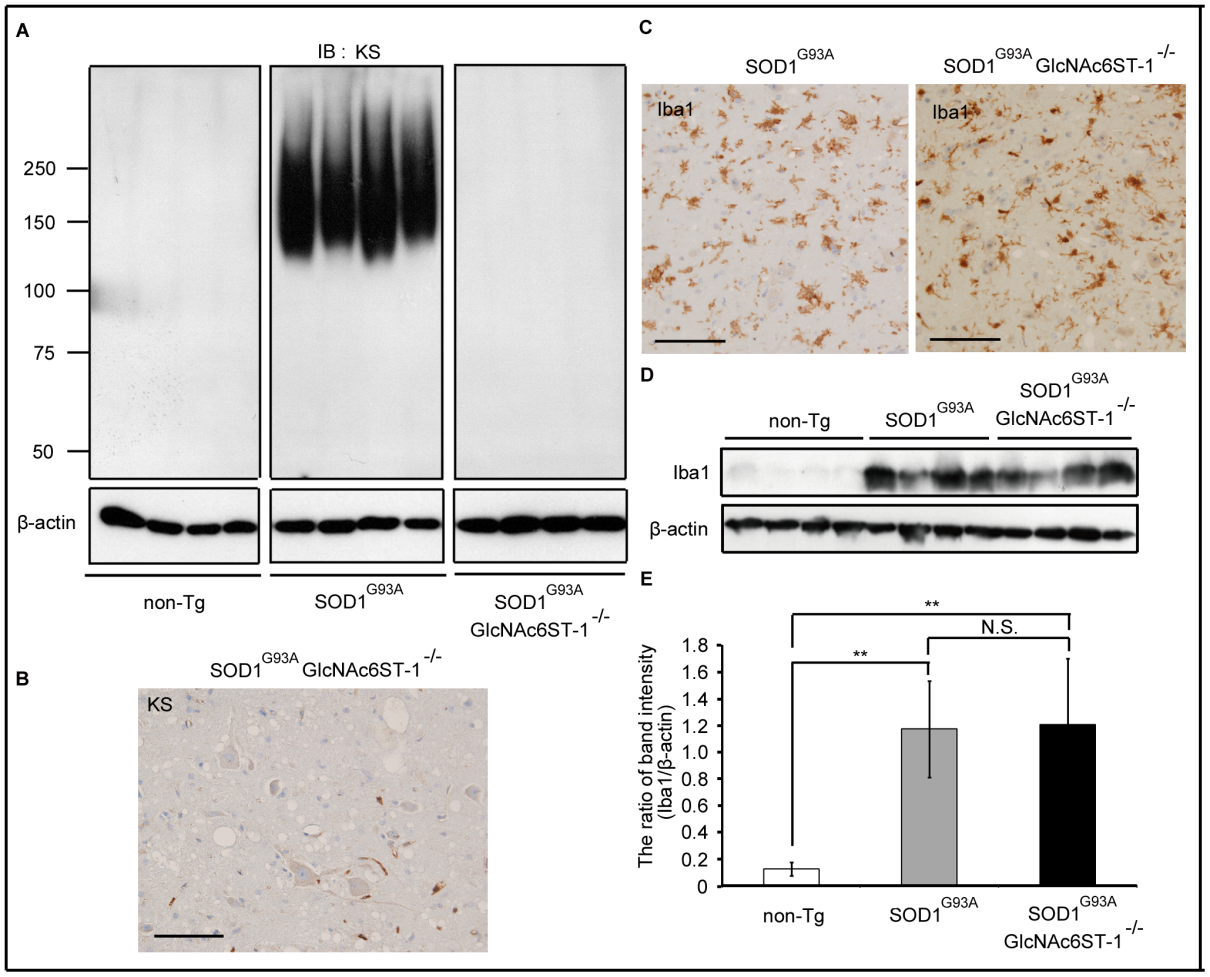
KS expression was attenuated in the SOD1^G93A^GlcNAc6ST-1^−/−^ mouse spinal cord. KS expression in the spinal cords of SOD1^G93A^ mice, age-matched non-Tg and SOD1^G93A^GlcNAc6ST-1^−/−^ mice at 24 weeks was examined by immunoblotting (A) and immunohistochemical (B) analysis. KS was not expressed in the spinal cords of SOD1^G93A^GlcNAc6ST-1^−/−^ mice. Bars, 200 µm. The number of microglia was examined by immunohistochemical (C) and immunoblotting (D) against Iba1 at 24 weeks. Bars, 200 µm. β-actin was used as the internal control (n = 4). E, The Iba1 band intensities were quantified by ImageJ. Error bars, SE. **p<0.01. N.S., not significant.

The finding that KS was expressed in microglia in both human and mouse ALS prompted us to ask whether KS had a biological role. Because the loss of KS in GlcNAc6ST-1^−/−^ mice is seen in the CNS (Uchimura et al., unpublished data) [15], SOD1^G93A^GlcNAc6ST-1^−/−^ mice are thought to be a suitable model to investigate the role of KS in ALS. Based on this idea, we generated SOD1^G93A^GlcNAc6ST-1^−/−^ mice, and compared their phenotype with that of SOD1^G93A^ mice. As expected, KS was not expressed in the spinal cords of SOD1^G93A^GlcNAc6ST-1^−/−^ mice (Figure 5A, B). Nevertheless, the microglia increased in number in the spinal cord to a similar extent in both SOD1^G93A^GlcNAc6ST-1^−/−^ and SOD1^G93A^ mice at 24 weeks (Figure 5C–E).

**Figure 6:**
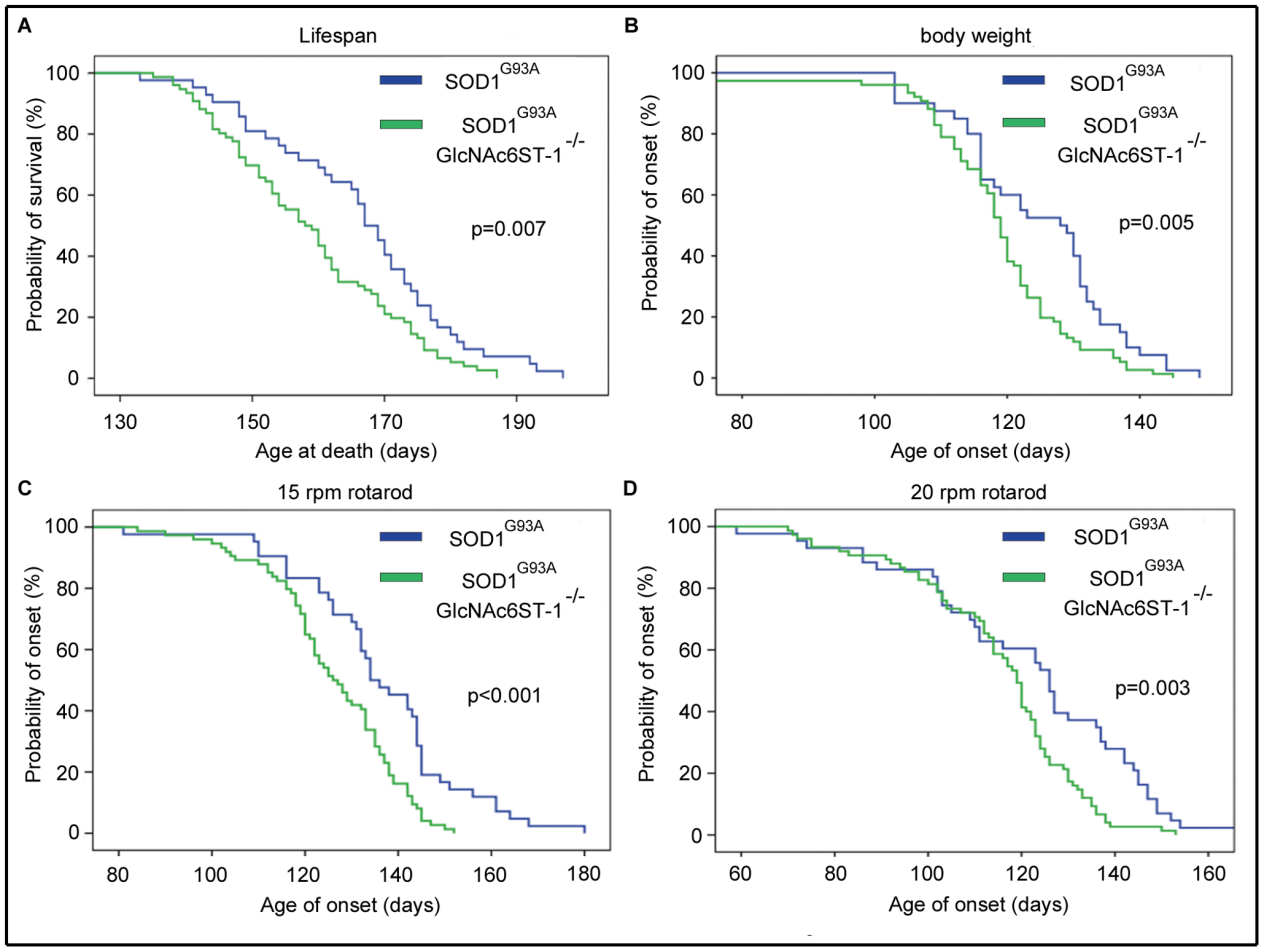
Ablation of KS in microglia accelerated the early phase pathogenesis in a mouse model. (A) The lifespan of SOD1^G93A^ was 164.7±18.2 days (n = 43), while that of SOD1^G93A^GlcNAc6ST-1^−/−^ was 158.7±13.2 days (n = 76) (p = 0.007). (B) Body weight loss of SOD1^G93A^ began at 124.7±12.2 days, while it began at 118.4±11.9 days in SOD1^G93A^GlcNAc6ST-1^−/−^ (p = 0.005). A decrease in rotarod performance at (C) 15 rpm and (D) 20 rpm of SOD1^G93A^ began at 141.4±18.2 days and 121.6±24.5 days, respectively. In SOD1^G93A^GlcNAc6ST-1^−/−^ mice, these onset times were accelerated to 125.2±14.2 (p<0.001) days and 112.5±18.1 days (p = 0.003), respectively.

To our surprise, a significant difference in lifespan was observed between these two genotypes. Lifespan was significantly decreased in SOD1^G93A^GlcNAc6ST-1^−/−^ mice (Figure 6A). Appearance of body weight loss was also accelerated in SOD1^G93A^GlcNAc6ST-1^−/−^ mice (Figure 6B). A decrease in rotarod performance at 15 rpm and 20 rpm also occurred earlier in SOD1^G93A^GlcNAc6ST-1^−/−^ mice (Figure 6C, D). Thus, ablation of KS in microglia resulted in acceleration of appearance of clinical symptoms and consequently shortened the lifespan in the mouse ALS model.

### KS-positive Microglia Consisted of CD86-positive and -negative Cells

**Figure 7:**
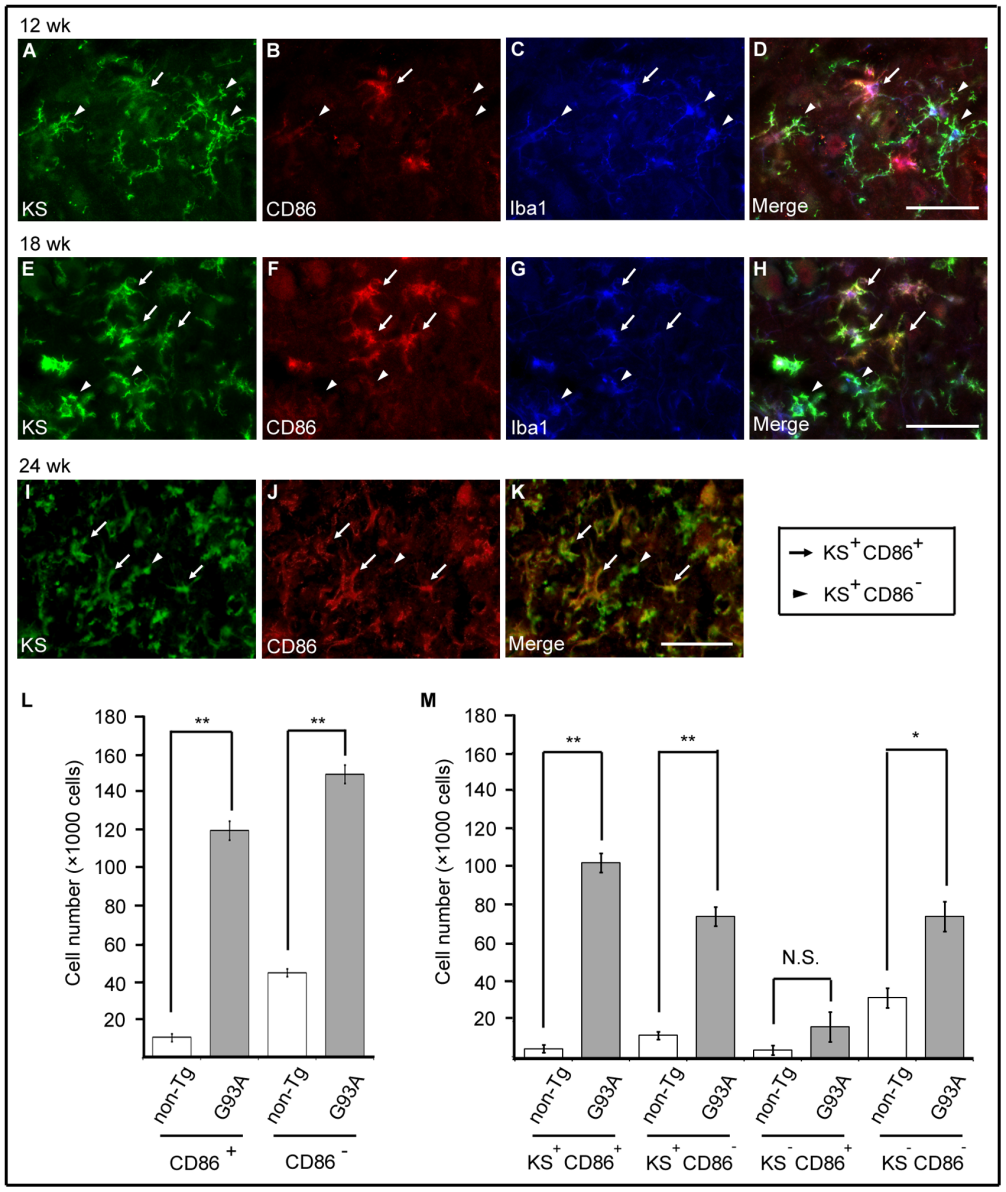
A subpopulation of KS-positive microglia was CD86-positive. The spinal cord sections of SOD1^G93A^ mice at 12, 18 and 24 weeks were stained with KS (A, E, I), M1 marker CD86 (B, F, J), and microglia marker Iba1 (C, G). The merged images are shown in (D, H, K). Arrows, KS^+^CD86^+^ microglia; arrowheads, KS^+^CD86^−^ microglia. Bars, 50 µm. Flow cytometric analysis for CD11b^+^ cells at 24 weeks. Summaries of CD86 expression (L) and expressions of CD86 and KS (M) are shown. Gray columns, SOD1^G93A^ mice; black columns, SOD1^G93A^GlcNAc6ST-1^−/−^ mice. Error bars, SE. **p<0.01, *p<0.05 (n = 3), N.S., not significance.

To investigate the mechanism underlying the shortened lifespan of SOD1^G93A^GlcNAc6ST-1^−/−^ mice, we further characterized KS-positive microglia. We compared the spatio-temporal expression of KS and CD86, a representative marker for M1 microglia, in SOD1^G93A^ mice at the early (12 weeks), body weight loss (18 weeks) and disease end stage (24 weeks) phases. KS began to be expressed at 12 weeks (Figure 7A). At this phase, although both KS^+^CD86^−^ and KS^+^CD86^+^ microglia existed, KS^+^CD86^−^ microglia were the major population in SOD1^G93A^ mice (Figure 7A–D). The proportion of KS^+^CD86^+^ microglia increased with disease progression, but KS^+^CD86^−^ microglia still existed (Figure 7E–K). Flow cytometric analysis for microglia, which were enriched by CD11b affinity beads from mice at 24 weeks, revealed that the numbers of both CD86^+^ and CD86^−^ cells were increased in SOD1^G93A^ mice (Figure 7L). Most of the CD86^+^ cells were KS^+^, while a significant portion of the CD86^−^ cells were KS^+^ (Figure 7M). Thus, the population of KS^+^CD86^−^ microglia was comparable with that of KS^+^CD86^+^ microglia in SOD1^G93A^ mice at 24 weeks (Figure 7M).

### The Transient Enhancement of M2 Markers was Diminished in SOD1^G93A^GlcNAc6ST-1^−/−^ Mice

**Figure 8:**
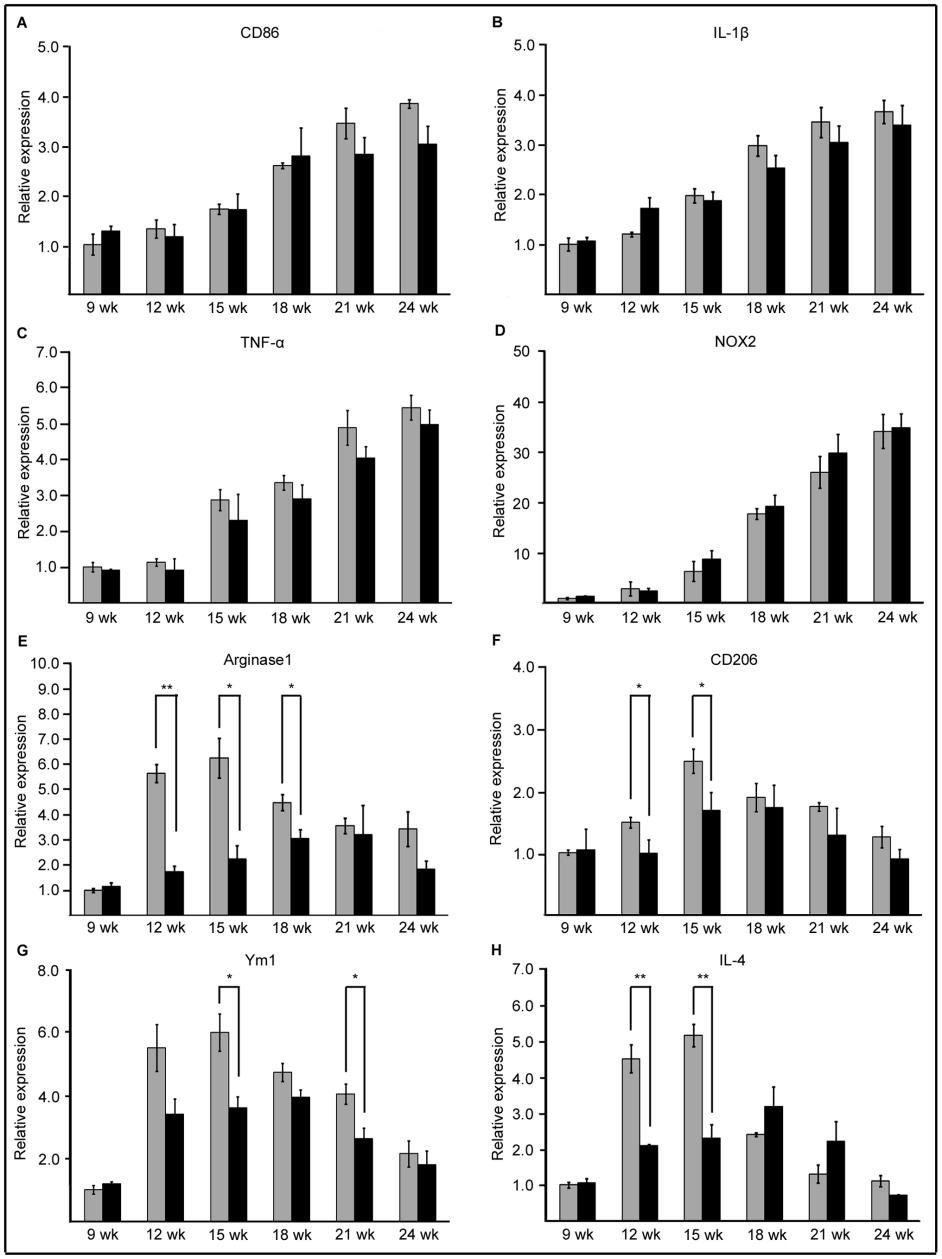
Transient enhancement of the expression of M2 markers was diminished in SOD1^G93A^GlcNAc6ST-1^−/−^ mice. The temporal mRNA expression profiles of M1 [CD86 (A), IL-1β (B), TNF-α (C) and NOX2 (D)] and M2 [Arginase1 (E), CD206 (F), Ym1 (G) and IL-4 (H)] markers were examined by quantitative RT-PCR. Gray columns, SOD1^G93A^ mice; black columns, SOD1^G93A^GlcNAc6ST-1^−/−^ mice. Error bars, SE. **p<0.01, *p<0.05 (n = 3).

Considering that the KS-positive microglia consisted of CD86-positive and -negative cells, we next examined the temporal expression profiles of M1 and M2 markers in the spinal cord. Expression of the M1 markers CD86, IL-1beta, TNF-alpha, and NOX2 was steadily increased as the disease progressed (Figure 8A–D). There was no difference in M1 marker expression between SOD1^G93A^ and SOD1^G93A^GlcNAc6ST-1^−/−^ mice (Figure 8A–D).

In contrast, the expression profiles of the M2 markers CD206, arginase1, Ym1 and IL-4 were significantly different between SOD1^G93A^ and SOD1^G93A^GlcNAc6ST-1^−/−^ mice based on a statistical analysis with two-way ANOVA (Figure 8E–H). Thus, the expressions of these genes were transiently enhanced at 12–15 weeks of age in SOD1^G93A^ mice (Figure 8E–H). This is the early phase before body weight loss, when KS began to be expressed in microglia (Figure 2). Notably, this enhancement of M2 marker expression observed in SOD1^G93A^ mice was strikingly attenuated in SOD1^G93A^GlcNAc6ST-1^−/−^ mice (Figure 8E–H).

**Figure 9:**
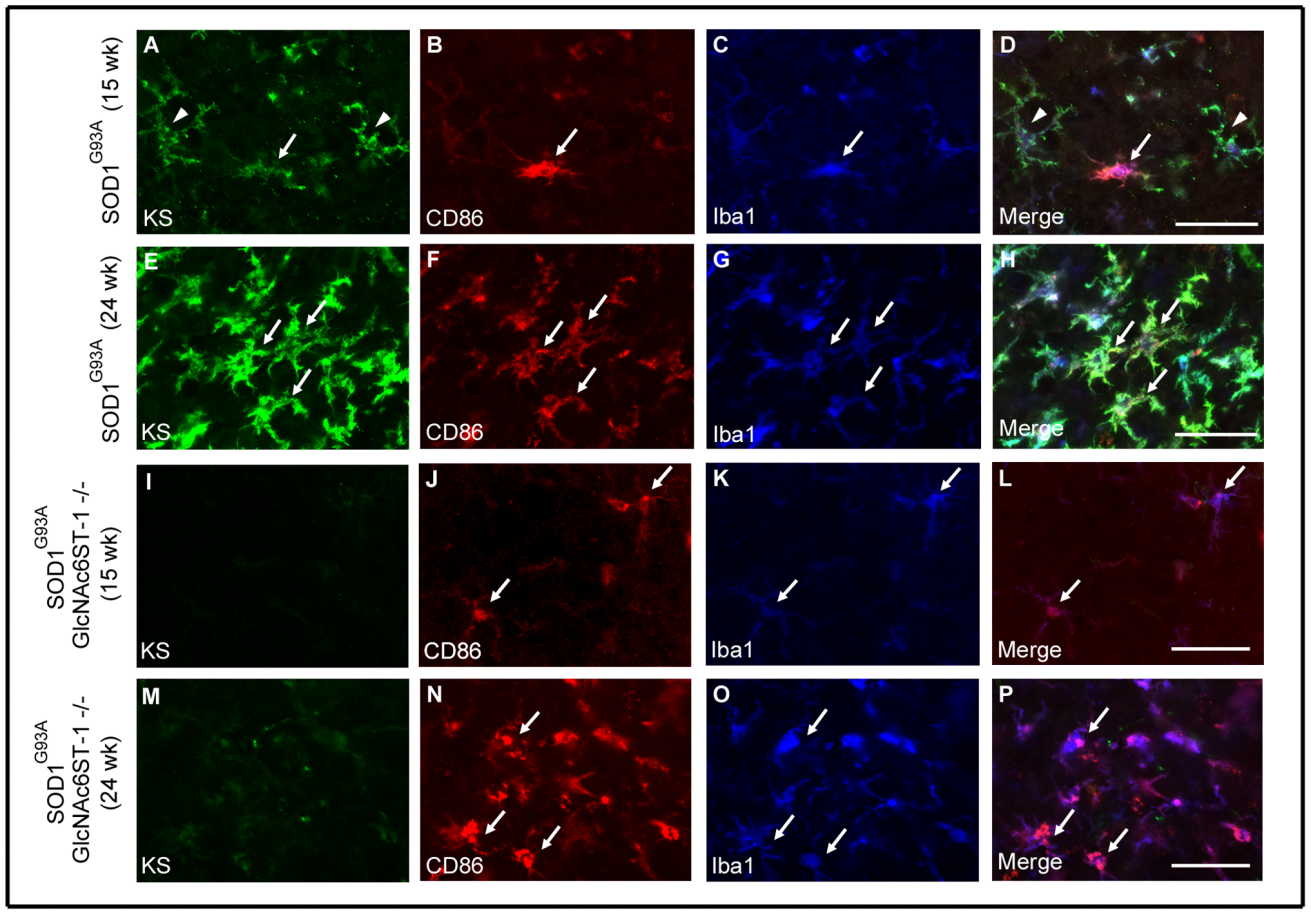
M1 microglia expanded as the disease progressed. The spinal cord sections of SOD1^G93A^ mice and SOD1^G93A^GlcNAc6ST-1^−/−^ mice at 15 and 24 weeks were stained with KS (A, E, I, M), M1 marker CD86 (B, F, J, N), and microglia marker Iba1 (C, G, K, O). Arrows in A-H, KS^+^CD86^+^ microglia; arrows in J-P, KS^–^CD86^+^ microglia; arrowheads, KS^+^CD86^−^ microglia. Bars, 50 µm.

**Figure 10:**
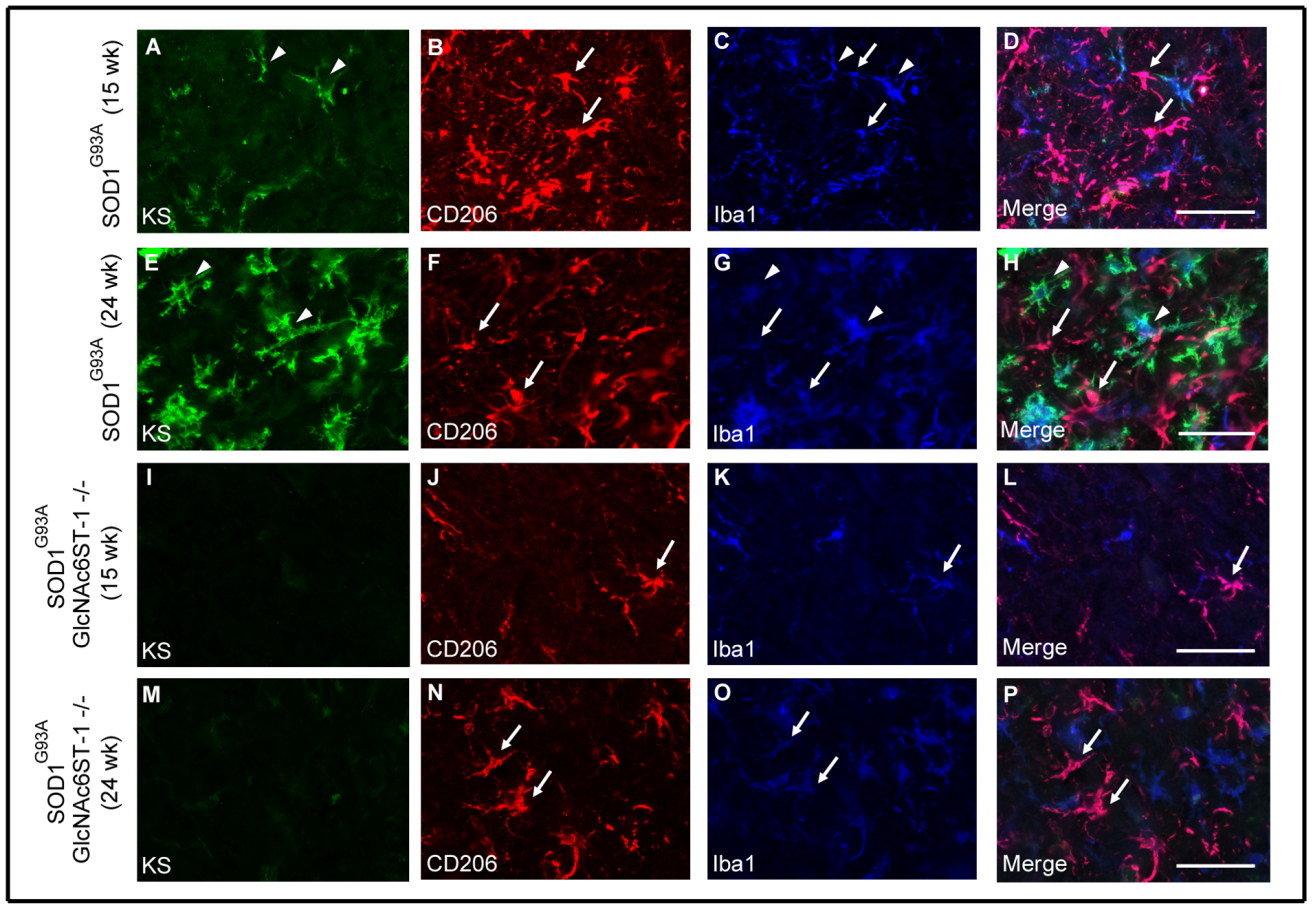
Expansion of M2 microglia during the early disease phase was suppressed in SOD1^G93A^GlcNAc6ST-1^−/−^ mice. The spinal cord sections of SOD1^G93A^ mice and SOD1^G93A^GlcNAc6ST-1^−/−^ mice at 15 and 24 weeks were stained with KS (A, E, I, M), M2 marker CD206 (B, F, J, N), and microglia marker Iba1 (C, G, K, O). Arrows, KS^–^CD206^+^ microglia; arrowheads, KS^+^CD206^−^ microglia. Bars, 50 µm.

We next examined the fate of M1 and M2 microglia during progression of the disease. M1 microglia (CD86-positive) consistently increased in number as the disease progressed from 12 weeks to 24 weeks in both SOD^G93A^ mice and SOD^G93A^GlcNAc6ST-1^−/−^ mice (Figure 7 and 9). In contrast, M2 microglia (CD206-positive) were abundant at 15 weeks and were continuously detectable until 24 weeks in SOD^G93A^ mice, whereas SOD^G93A^GlcNAc6ST-1^−/−^ mice showed markedly less M2 microglia at 15 weeks (Figure 10). These profiles were almost consistent with those of marker expression shown in Figure 8.

**Figure 11:**
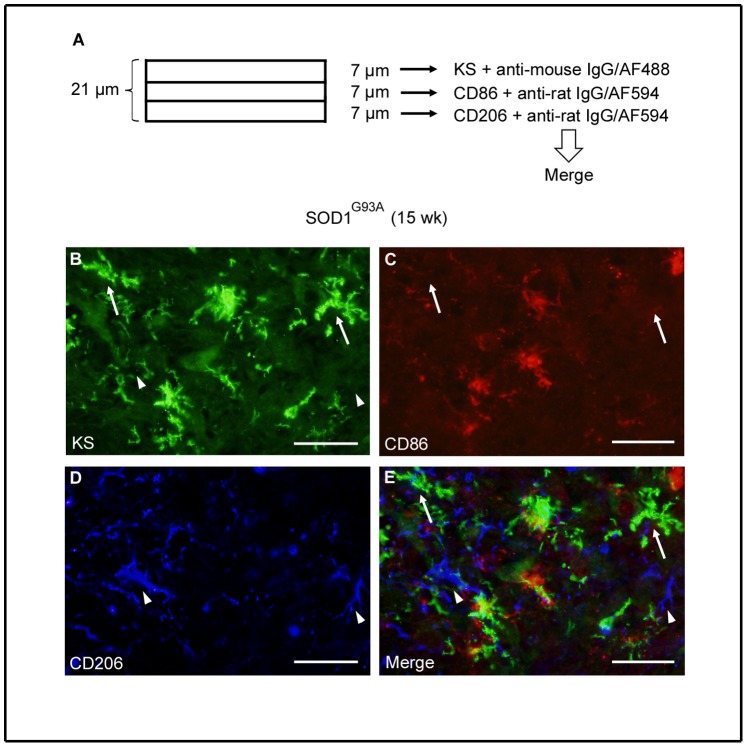
CD206 was expressed in a subpopulation of microglia distinct from KS+CD86- cells. Sequential thin sections were subjected to immunohistochemical analyses (A). The spinal cord sections of SOD1^G93A^ mice at 15 weeks were stained with KS (B), CD86 (C) and CD206 (D). Arrows, KS^+^CD86^−^ microglia; arrowheads, KS^–^CD206^+^ microglia. Bars, 50 µm.

As a majority of KS-positive microglia were CD86-negative at 12 weeks (Figure 7A–D), these KS^+^CD86^−^ microglia could be a major source of future polarized cells. We asked if KS^+^CD86^−^ microglia were M2 microglia (CD206-positive) at 15 weeks. Immunohistochemical analysis using sequential thin sections revealed that KS^+^CD86^−^ microglia did not express CD206 at 15 weeks, while CD206-positive cells were KS-negative (Figure 11). Our data collectively suggest that KS^+^CD86^−^ microglia could be directly polarized to KS^–^CD206^+^ microglia or indirectly influence the polarization of other types of cells to KS^–^CD206^+^ microglia as the disease progresses, although other pathways of M2 polarization cannot be excluded.

## Discussion

Striking progress has been made in our understanding of neurobiology in the last several decades. Most of the advances have been realized by focusing on proteins as the main players in neurobiological mechanisms. However, considering that biopolymers consist of three main classes, i.e., polynucleotides (DNA and RNA), polypeptides (proteins) and polysaccharides (sugar chains), important biological functions are also expected to be attributable to sugar chains. In addition, most proteins are modified with sugar chains. It has recently been accepted that CS is a potent inhibitor of axonal regeneration/sprouting and synaptic plasticity [12], [13], and several candidate receptors for CS, such as PTPsigma and Nogo receptor 1 and 3, have been postulated [18], [19]. Although these findings have promoted the emerging research area of glyco-neurobiology, the roles of sugar chains in the nervous system remain largely unknown. The present study is the first to demonstrate an indispensable role of a sugar chain in the pathogenesis of a neurodegenerative disease.

We found in the current study that KS-positive microglia progressively expand in SOD1^G93A^ mice (Figure 2). Based on this observation, we initially expected that SOD1^G93A^GlcNAc6ST-1^−/−^ mice might show a less lethal phenotype compared to SOD1^G93A^ mice. However, we found that the ablation of KS in microglia resulted in acceleration of clinical symptoms (body weight loss and decreased rotarod performance) and consequently shortened the lifespan (Figure 6). This phenotype is associated with the attenuation of the transient upregulation of M2 microglia markers, e.g., arginase 1, CD206, Ym1 and IL-4, during the early disease phase (12–15 weeks) before body weight loss in SOD1^G93A^GlcNAc6ST-1^−/−^ mice (Figure 8). Considering that these M2 markers are reported to be closely associated with anti-inflammatory functions [7], [8], [10], [11], our data collectively suggest that KS plays an indispensable, suppressive role in the early phase pathogenesis of ALS. Furthermore, consistent with the profiles of marker expression, M2 microglia (CD206-positive) are markedly less during the early disease phase in SOD1^G93A^GlcNAc6ST-1^−/−^ mice compared with SOD1^G93A^ mice, while the consistent increase of M1 microglia (CD86-positive) is comparable in these two genotypes (Figure 7, 9, 10). As a majority of KS-positive microglia are CD86-negative (KS^+^CD86^−^ microglia) during the early disease phase, these microglia probably play an indispensable role (Figure 7). Although it is speculated that the suppressive role of KS in the early pathogenesis is mediated through the promotion of microglia polarization to an anti-inflammatory state, there might be other possibilities, e.g., the KS expressed by microglia might influence the functions of other cell types.

Our study raises the question of whether the function of KS can be generalized to that of other glycosaminoglycans, such as CS, in the pathogenesis of neurodegenerative diseases. There have been only a few reports in this field. Chondroitin sulfate proteoglycans (CSPGs) (i.e., neurocan, versican, phosphacan, and decorin) accumulate in the microenvironment of spinal motoneurons in ALS transgenic rats [20], [21]. These CSPGs are mainly produced by activated astrocytes, but their function is unclear. Our current study highlighted the importance of KS expressed in a subpopulation of microglia. With regard to microglia polarization, it is interesting that microglia/macrophages are activated by CSPGs located in the glial scar after neuronal injury, and polarized into a beneficial state which produces IGF-1 and BDNF [22], [23]. Verification of molecular interactions involving KS and CS may shed light on the pathogenesis of ALS and other neurodegenerative diseases. In addition, because the expression of KS synthesis enzymes is concomitantly upregulated (Figure 3), studies on the regulatory mechanism of the expression of these enzymes may provide clues to effective therapy or diagnostic methods for ALS.

## Materials and Methods

### Mice

The animal experiments described in this article were performed in accordance with protocols approved by the Animal Care and Use Committee of Nagoya University Graduate School of Medicine. All animals were treated and cared for in accordance with the Nagoya University School of Medicine Guidelines pertaining to the treatment of experimental animals. SOD1^G93A^ transgenic mice, which carry the G93A mutant form of the human SOD1 (B6.Cg-Tg [SOD1-G93A] 1Gur/J line), were purchased from the Jackson Laboratory. Maintenance of these mice and their genotyping were done as described previously [24]. GlcNAc6ST-1^−/−^ mice were produced using D3 embryonic stem cells and ordinary gene-targeting as described previously [14]. GlcNAc6ST-1^+/−^ mice obtained after backcrossing with C57BL/6J for more than 11 generations were interbred and these mice were used for mating. The sequences of the primers used for genotyping are listed in Table 1.

**Table 1:** The primer sequences determined using genotyping of mice.

Target gene	Direction	Primer sequence
SOD1 F	Forward	CATCAGCCCTAATCCATCTGA
	Reverse	CGCGACTAACAATCAAAGTGA
GlcNAc6ST-1 F	Forward	AAGCCTACAGGTGGTGCGAA
	Reverse	CAGGACTGTTAACCCGCTCA
Neo GlcNAc6ST-1	Forward	AGCGTTGGCTACCCGTGATA
	Reverse	GCCAAAAGTGATCACCTCGT
GAPDH	Forward	ACTCACGGCTTCAACG
	Reverse	CCCTGTTGCTGTAGCCGTA

### Human ALS Samples

Specimens of lumbar spinal cord (L4–L5 segments) from ALS cases were obtained. Among the specimens examined, those with preserved expression of Iba1 (2 familial and 3 sporadic ALS cases) were subjected to further evaluation of KS expression. The diagnosis of ALS was confirmed by the El Escorial diagnostic criteria published by the World Federation of Neurology and by histopathologic findings, particularly the presence of Bunina bodies. The molecular diagnosis was negative for SOD1, FUS/TLS, TDP-43 and OPTN in familial ALS cases. The collection of autopsied human tissues, their use for this study, and consent procedure were approved by the Ethics Committee of Nagoya University Graduate School of Medicine, and written informed consent was obtained from the patients’ next-of-kin.

### Lifespan Experiments

Mice were observed daily for survival. An investigator who was blinded to the genotype of mice measured the body weight and motor performance. Both measurements were started at the age of 60 days, and the body weight of mice was measured every 5 days. Testing of motor function using a Rota-rod Treadmill for mice (MK-610A; Muromachi,) was performed once a week. Each weekly session consisted of three trials on the constantly rotating setting at a speed of 15 and 20 rpm. The time remaining on the rotor during a 3 min period was recorded. Mice were judged to have failed the test when, on average in three separate trials, they fell off the rotarod before 2 min. Time of disease onset was retrospectively determined as the time when mice reached peak body weight or the time when mice began to fail the rotarod test. The time of end stage was determined by paralysis so severe that the animal could not right itself within 20 seconds when placed on its side, an endpoint frequently used for SOD1 mutant-expressing mice [25]. Lifespan was defined by the duration between the birth and the end stage.

### Immunohistochemistry

Mice were deeply anesthetized with diethylether and transcardially perfused with phosphate buffered saline (PBS) followed by 4% paraformaldehyde (PFA) in 0.1M phosphate buffer (Wako). Isolated lumbar spinal cords were fixed with 4% PFA overnight at 4°C, and cryoprotected by 20% sucrose in 0.1M phosphate buffer at 4°C during the subsequent night. The tissue samples were embedded in Tissue-Tek O.C.T. compound (Sakura Finetek) and quickly frozen by liquid nitrogen. Frozen tissues were cut into 10 µm sections on a cryostat (CM1800; Leica Instruments) and collected on MAS-coated glass slides (Superfrost; Matsunami Glass).

The sections were washed in PBS. After blocking with 3% bovine serum albumin (BSA) in PBS containing 0.1% Triton X-100 for 30 min at room temperature, the sections were incubated at 4°C overnight with the following primary antibodies: mouse anti-KS (1∶500, clone 5D4; Seikagaku Corporation), rabbit anti-Iba1 (1∶500; Wako), rabbit anti-GFAP (1∶1000; Sigma), rabbit anti-NG2 (1∶500; Millipore), rat anti-CD86 (1∶200; BD Biosciences), and rat anti-CD206 (1∶200; AbD Serotec). After three rinses with PBS, the sections were incubated for 60 min at room temperature with the following secondary antibodies: AlexaFluor 594 conjugated anti-mouse IgG (Invitrogen), AlexaFluor 488 conjugated anti-rabbit IgG (Invitrogen), AlexaFluor 488 conjugated Donkey anti-mouse IgG (Invitrogen), AlexaFluor 594 conjugated Donkey anti-rat IgG (Invitrogen), and AlexaFluor 647 conjugated Donkey anti-rabbit IgG (Invitrogen). For paraffin sections, isolated lumbar spinal cords were fixed with 4% PFA overnight at 4°C, dehydrated with 70% ethanol, and embedded in paraffin. These tissues were cut into 5 µm sections. Immunohistochemistry analysis was performed using a Ventana DISCOVERY system (Ventana Medical Systems). Sections were incubated overnight at 4°C with the following antibodies: mouse anti-KS (1∶500; clone 5D4), and rabbit anti-Iba1 (1∶500). After three rinses with PBS, the sections were incubated with horseradish peroxidase (HRP)-conjugated secondary antibodies (1∶5000; Jackson ImmunoResearch). Binding antibodies were visualized by using a Ventana DAB Map kit (Ventana Medical Systems).

### SDS-PAGE and Immunoblotting

Mice were deeply anesthetized with diethylether and perfused transcardially with PBS. Each isolated lumbar spinal cord was homogenized in lysis buffer (1% Triton X-100 and 1% protease inhibitor cocktail (Nacalai Tesque) in PBS) for 60 sec at 4°C. The homogenates were centrifuged at 20000×*g* for 15 min, and the concentration of the soluble proteins was measured by Bradford protein assay using a Protein Quantification Kit (Dojindo). The soluble proteins were digested with protease-free chondroitinase ABC (1 U/ml; Seikagaku Corporation) in 50 mM Tris-acetate buffer (pH 8.0) for 2 hr at 37°C. The digested and non-digested samples were mixed with 4×loading buffer (0.25 M Tris-HCl, 20% mercaptoethanol, 8% SDS, 20% sucrose, 0.008% bromophenol blue; pH 6.8) and boiled for 5 min. SDS-PAGE was performed in 6% and 15% gels. After transferring the proteins to a polyvinylidene difluoride (PVDF) membrane (Hybond-P; GE Healthcare) and blocking it with PBS-T containing 5% skim milk for 1 hr at room temperature, the membranes were incubated with mouse anti-KS (1∶2000; clone 5D4), rabbit anti-Iba1 (1∶1000; Wako), and mouse anti-β-actin (1∶10000; Sigma). After three washes with 5% skim milk in PBS-T, the membranes were incubated with the HRP-conjugated secondary antibodies for 30 min at room temperature. Binding antibodies were visualized by an ECL Plus kit (GE Healthcare).

### Quantitative RT-PCR

Total RNA was extracted from the lumbar spinal cords using an RNeasy Lipid Tissue kit (Qiagen) according to the manufacturer’s recommendations. The cDNA was prepared from 1 µg of total RNA by using a Transcriptor First Strand cDNA Synthesis Kit (Roche) following the standard protocols. Quantitative PCR was performed on a StepOne (Applied Biosystems) or Mx3000P (Agilent Technologies) using Taqman Probes or SYBR Green (Agilent Technologies), respectively. Samples were subjected to 40 cycles of amplification at 95°C for 15 sec and 60°C for 1 min, after holding at 50°C for 2 min and 95°C for 10 min. Relative expression was calculated using the 2-^(Ct experimental sample – Ct internal control sample (GAPDH))^ method. The Taqman probes used for the quantitative RT-PCR of KS synthesis enzymes were as follows; Mm00507533_m1, Mm00490018_g1, Mm00488783_s1, Mm00517342_m1, Mm00491466_m1, Mm00480087_m1, and Mm00517855_m1. The sequences of primers used for the quantitative RT-PCR of M1 and M2 markers are listed in Table 2.

**Table 2:** The primer sequences using quantitative RT-PCR of M1 and M2 markers.

Target gene	Direction	Primer sequence
CD86	Forward	ACGATGGACCCCAGATGCACCA
	Reverse	GCGTCTCCACGGAAACAGCA
IL-1β	Forward	CCTGCAGCTGGAGAGTGTGGAT
	Reverse	TGTGCTCTGCTTGTGAGGTGCT
TNF	Forward	AGCCCACGTCGTAGCAAACCAC
	Reverse	AGGTACAACCCATCGGCTGGCA
NOX2	Forward	TCTCAGGGGTTCCAGTGCGTGT
	Reverse	TGTGGATGGCGGTGTGCAGT
Arginase1	Forward	TTAGGCCAAGGTGCTTGCTGCC
	Reverse	TACCATGGCCCTGAGGAGGTTC
CD206	Forward	TCAGCTATTGGACGCGAGGCA
	Reverse	TCCGGGTTGCAAGTTGCCGT
Ym1	Forward	ACCCCTGCCTGTGTACTCACCT
	Reverse	CACTGAACGGGGCAGGTCCAAA
IL-4	Forward	TGGGTCTCAACCCCCAGCTAGT
	Reverse	TGCATGGCGTCCCTTCTCCTGT

### Flow Cytometry

The spinal cords obtained from non-Tg and SOD1^G93A^ mice at 24 weeks were dissociated with 5 mg/ml collagenase in HBSS at 37°C for 45 min, and filtered by using 70 µm cell strainers. Immune cells were separated by centrifugation using 38% Percoll in PBS at 2000×*g* for 20 min. The immune cells were then suspended with PBS containing 0.5% BSA and 2 mM EDTA and incubated with CD11b magnetic beads (Milteny Biotec GmbH) at 4°C for 15 min. Fcγ receptors on CD11b-enriched cells were blocked by incubation with anti-CD16/CD32 antibody (1∶400; BD Biosciences) at 4°C for 15 min. Then, the cells were stained by PE-Cy^TM^7 conjugated anti-CD86 antibody (2 µg/sample; BD Biosciences) and anti-KS antibody (2 µg/sample) which was labeled with a Zenon Alexa 405 mouse IgG1 labeling kit (Invitrogen) at 4°C for 30 min, and analyzed with FACS Canto II, FACSDiva (Becton Dickinson) and FlowJo (Tree Star).

### Statistical Analysis

An unpaired Student’s two-tailed *t*-test was used for analyzing the mRNA expression of the KS synthesis enzymes. The Kaplan Meier method (log-rank test) was used for comparing the lifespan and disease onset of mice of each genotype. Mann Whitney’s U test was used for analyzing disease duration. Two-way ANOVA was used to evaluate the difference in the time course of M1 and M2 markers between two groups. We subsequently performed the unpaired Student’s two tailed *t*-test at each week. In all statistical analyses, values of p<0.05 were considered to indicate significance. The statistical analysis was performed using SAS 9.3 (SAS Institute Inc.) and SPSS (SPSS Inc.) software. The investigators performing the statistical analyses were blinded to the group assignments in all procedures.
